# An Electrical Stimulation Culture System for Daily Maintenance-Free Muscle Tissue Production

**DOI:** 10.34133/2021/9820505

**Published:** 2021-04-08

**Authors:** Yoshitake Akiyama, Akemi Nakayama, Shota Nakano, Ryuichiro Amiya, Jun Hirose

**Affiliations:** ^1^Faculty of Textile Science and Engineering, Shinshu University, 3-15-1 Tokida, Ueda, Nagano, Japan; ^2^Department of Biomedical Engineering, Shinshu University, 3-15-1 Tokida, Ueda, Nagano, Japan; ^3^Tech Alpha, 649-1 Ohtsuka, Hachioji, Tokyo, Japan

## Abstract

Low-labor production of tissue-engineered muscles (TEMs) is one of the key technologies to realize the practical use of muscle-actuated devices. This study developed and then demonstrated the daily maintenance-free culture system equipped with both electrical stimulation and medium replacement functions. To avoid ethical issues, immortal myoblast cells C2C12 were used. The system consisting of gel culture molds, a medium replacement unit, and an electrical stimulation unit could produce 12 TEMs at one time. The contractile forces of the TEMs were measured with a newly developed microforce measurement system. Even the TEMs cultured without electrical stimulation generated forces of almost 2 mN and were shortened by 10% in tetanic contractions. Regarding the contractile forces, electrical stimulation by a single pulse at 1 Hz was most effective, and the contractile forces in tetanus were over 2.5 mN. On the other hand, continuous pulses decreased the contractile forces of TEMs. HE-stained cross-sections showed that myoblast cells proliferated and fused into myotubes mainly in the peripheral regions, and fewer cells existed in the internal region. This must be due to insufficient supplies of oxygen and nutrients inside the TEMs. By increasing the supplies, one TEM might be able to generate a force up to around 10 mN. The tetanic forces of the TEMs produced by the system were strong enough to actuate microstructures like previously reported crawling robots. This daily maintenance-free culture system which could stably produce TEMs strong enough to be utilized for microrobots should contribute to the advancement of biohybrid devices.

## 1. Introduction

Biohybrid robotics that integrates living components with synthetic structures is currently one of the most challenging fields of robotics [1]. Among biohybrid robotics, muscle-actuated biohybrid devices have attracted much attention of researchers in not only mechanical engineering but also in bioengineering and material chemistry [2]. Toward practical use of muscle-actuated devices, low-labor production of tissue-engineered muscles (TEMs) is one of the key technologies. This study develops and demonstrates daily maintenance-free production of TEMs from immortal myoblast cells using an electrical stimulation function.

Animal muscular systems have evolved by natural selection over several billion years. Compared to state-of-the-art artificial actuators, natural muscle has several distinguishing and desirable advantages, such as its high-energy conversion efficiency, its independency from electrical or fossil fuel energy supplies, its softness and flexibility, and its capability for self-repair [3]. Many muscle-actuated devices have been reported in the past decade such as pumping devices [4, 5], manipulators [6, 7], crawling robots [8–10], and swimming robots [11–14]. Studies on biohybrid devices have been summarized in several recently published review articles [15–18].

Most studies on biohybrid devices have utilized mammalian muscle cells as actuators. Among the three major categories of mammalian muscles, heart, skeletal, and smooth muscles, heart-derived muscle cells, also called cardiomyocytes, are utilized most frequently as actuators. Cardiomyocytes enable the fabrication of autonomously working devices without any external control owing to their autonomy. On the other hand, it is difficult to control, in particular, to suspend, autonomous contractions of cardiomyocytes. With skeletal muscle, users have to stimulate them constantly, but the contractile state and force can be controlled precisely by adjusting the external stimuli [19]. That makes skeletal muscle-actuated devices advantageous for user-controlled manipulation [7, 20]. Therefore, skeletal muscle should be the most versatile substitute for mechanical motors and actuators.

A long-term culture from 1 to 3 weeks is necessary to obtain contractile TEMs [21]. Skeletal muscle tissues consist of large amounts of myofibers that are formed by differentiation and maturation after the fusion of undifferentiated immature cells known as myoblasts [22]. The fusion process starts after myoblasts sufficiently proliferate to nearly a confluent state. And fused myoblasts, called myotubes, mature to myofibers. During the culturing period of several weeks, the medium must be replaced every one or two days. In contrast, popular actuators like electromagnetic motors are mass-produced at low-labor cost by an automated production line [23]. Therefore, daily maintenance-free production of TEMs which enables low-labor cost should be an important step toward the practical use of TEM-actuated devices.

To obtain a contractile force at the milli-Newton level for biohybrid devices, muscle cells have been reconstructed and utilized as TEMs [7, 20, 24]. Single muscle cells can generate only around 10 *μ*N [25], which is not sufficient to move structures of submillimeter size. In general, TEMs are obtained by culturing muscle cells in hydrogel scaffolds such as collagen and fibrin gel supplemented with Matrigel. To enhance the contractile force of TEMs, electrical pulse stimulation is frequently used as a surrogate for electrical pulses from nerves. Denervated muscle, owing to injury or disease, is known to cause myoatrophy and will degrade rapidly [26]. As well as denervation in vivo, TEMs cannot develop sufficiently under static culture conditions without any stimulation. The same as for two-dimensional (2D) muscle cell culture, the production system of TEMs should be equipped with an electrical stimulation function. Many groups have used commercially available electrical stimulators [27–29], and others have developed original ones [30–33].

A common problem of electrical stimulation is the need to replace medium every day or every other day to remove harmful byproducts of electrolysis that can damage the TEMs being grown. The present study offers a way to eliminate daily maintenance in constructing TEMs by introducing an automatic medium replacement function. We develop the daily maintenance-free culture system equipped with both electrical stimulation and medium replacement functions. In addition, the TEM transfer process from the gelation chamber to the electrical stimulation chamber depends on delicate manual operations, and TEMs may be easily damaged. The developed system can conduct gelation of TEMs and electrical stimulation to TEMs in the same chamber just by removing the insert for gelation. To avoid ethical issues, C2C12 cells [34] as immortal myoblast cells are cultured in collagen gel using the culture system. The contractile force of TEMs is evaluated with a newly developed microforce measurement system. Using these two systems, we also examine electrical stimulation conditions to improve TEMs based on the contractile force.

## 2. Materials and Methods

### 2.1. Experimental and Technical Design

**Figure 1:**
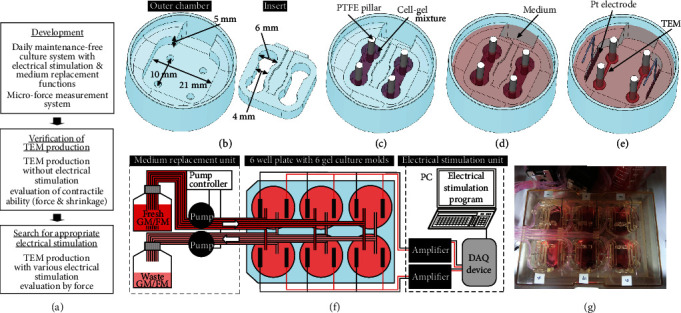
Schematic showing perspective views of the gel culture procedures in the developed system. (a) The research design flow. (b) The outer chamber and the gelation insert with their main dimensions. (c) The mold was filled with cell-gel mixture. Before filling, four PTFE pillars were attached to the mold. (d) Setup for gel culture. After gelation, the cell-gel mixture was overlaid with medium. (e) Setup for culture with electrical stimulation. The insert was removed and a pair of Pt electrodes were placed along the walls of the outer chamber. (f) Schematic illustration of the whole system. The medium replacement unit was a commercially available one. In the electrical stimulation unit, bipolar pulses generated by the DAQ device were amplified and applied to TEMs in the gel culture molds. (g) Photo of the six gel molds in the 6-well plate connected via silicone tubes with the pumps of the medium replacement unit. Pt electrodes were fixed in place with the lid of the 6-well plate and connected via metal wires with the electrical stimulation unit (not shown).

Our research with the goal of developing the daily maintenance-free culture system equipped with both electrical stimulation and medium replacement functions was performed in the order according to Figure 1(a). First, the electrical stimulation culture function was implemented. To measure the contractile force of TEMs, the microforce measurement system was also developed. Then, TEMs were produced without electrical stimulation by the culture system. The contractile ability of the TEMs was confirmed by measuring the contractile force and the shrinking distance. Finally, various electrical stimulations were applied to TEMs, and the conditions of the most appropriate stimulation were explored based on the obtained contractile force.

### 2.2. Gel Culture of Muscle Cells to Produce TEMs

Mouse myoblast cells C2C12 obtained from the American Type Culture Collection (ATCC) were cultured in growth medium (GM) for proliferation and in fusion medium (FM) for differentiation. Dulbecco's Modified Eagle's Medium (DMEM) supplemented with 10% fetal bovine serum and 1% antibiotics (06168-34, Nacalai Tesque) was used as GM and DMEM containing 50 ng/mL insulin-like growth factor 1(IGF-1) (100-11, Pepro Tech) and supplemented with 7% horse serum and 1% antibiotics was used as FM. TEMs were formed by culturing C2C12 cells in collagen gel in a similar manner to our previous study [35]. In brief, 2520 *μ*L of type I collagen solution, 315 *μ*L of ×10 concentrated DMEM, and 315 *μ*L of buffer solution for reconstitution were stirred on ice. These reagents were obtained from Nitta Gelatin. Then, 430 *μ*L of Matrigel (354234, Corning) was added and this mixture was also stirred on ice. Finally, 1928 *μ*L of C2C12 cell suspension was added at 5 × 10^6^ cells/mL as the final concentration and stirred well. The cell-gel mixture obtained by the described process was immediately poured into a gel culture mold.

### 2.3. Gel Culture Molds for TEMs

Figure 1(b) shows the gel culture mold consisting of an outer chamber and an insert. To provide high productivity, the outer size of the mold was designed to fit into a 6-well plate, and culturing could be done in six gel culture molds simultaneously by placing them into the 6-well plate. The gel culture molds were fabricated by milling polytetrafluoroethylene (PTFE) sheet with a numerical control machine (PRODIA-M45, Modia Systems). Each mold had two indentations (volume, *~*370 *μ*L) in which a TEM could be formed. In order to maintain the TEMs against spontaneous shrinkage of the gel, PTFE pillars *~*2 mm in diameter and *~*15 mm in length were fixed into place by inserting them into holes at the bottom of the indentations.

The steps to produce TEMs were as follows. First, the cell-gel mixture was poured into the indentations and incubated for 1.5 h at 37°C in a humidified atmosphere of 5% CO_2_ (Figure 1(c)). Then, the cell-gel mixture was overlaid with GM and the TEMs were cultured for 3 days (Figure 1(d)). After the insert was gently removed using tweezers, GM was replaced by FM. Then electrical stimulation was started and continued for 10 days (Figure 1(e)). FM was replaced every day during the stimulation.

### 2.4. Numerical Analysis of Electrical Field Generated by Electrical Stimulation

The electrical field generated by the electrical stimulation was analyzed with finite element method software (COMSOL Multiphysics ver 5.5). We assumed that the regions for the medium and TEM were a homogenous conductive material the same as the saline with 16 mS/cm. This is because the electrical conductivity of TEM depends on the frequency and the measurement method and varies from 0.05 to 7 mS/cm [36]. In addition, the TEM electrical conductivity should be nearly as high as that of the saline initially and be decreasing over time since the TEM was just a cell-gel mixture initially and it gradually differentiated into the muscle tissue with lower conductivity than the saline. The electrical field was calculated based on Ohm's equation: *J* = *s* *E*, where *J* is electrical current density vector, *s* is electrical conductivity, and *E* is electrical field density vector. The simulation was performed with a 2D model at the plane 1 mm from the bottom of the culturing area for TEMs.

### 2.5. Electrical Stimulation Culture Function

Figure 1(f) is a schematic illustration showing how we implemented the automatic electrical stimulation function using six gel culture molds, a medium replacement unit, and an electrical stimulation unit. Six gel culture molds were individually placed in the wells of a 6-well plate (3810-006, Iwaki). As each mold produced two TEMs, 12 TEMs in total were formed. As the medium replacement unit, a commercial automatic medium replacement device (CEME-0102, Takasago Electric) was used. The device removed almost all the GM or FM and then added about 3.5 mL of medium. The electrical stimulation unit applied electrical stimulation to TEMs in the gel culture mold. The bipolar pulses were generated by a data acquisition (DAQ) device (USB-6001, NI) with homemade software written in a graphical programing software (LabVIEW, NI). The pulses were amplified with bipolar amplifiers (BWA25-1, Takasago) and were applied to TEMs through a pair of Pt electrodes 5 × 10 mm^2^, which were manually placed along the walls in parallel to the TEMs as shown in Figure 1(g). Through the study, bipolar pulses ±4 V and 4 ms in width were applied to TEMs in order to inhibit the electrolysis as much as possible. Furthermore, FM was replaced every day during electrical stimulation to inhibit the electrolysis.

### 2.6. Contractile Force Measurement

**Figure 2:**
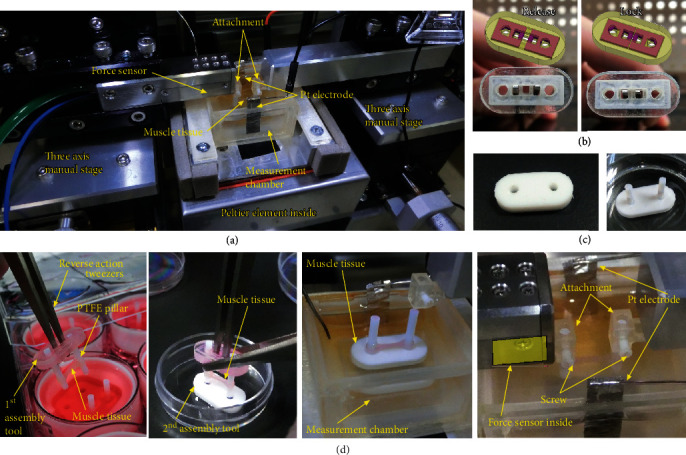
Force measurement of TEMs. (a) Photo showing the center of the force measurement system. (b) The first assembly tool. The magenta parts were not connected to the yellow part so that former parts moved freely inside the space. (c) The second assembly tool was fabricated by drilling two holes to hold the PTFE pillars. (d) Photo images for the steps to position the TEM.

Figure 2(a) shows the microforce measurement system, which was newly developed for this study. The system was composed of a force sensor, an amplifier, a temperature control unit, two microscopes, 3-axis manual stages, a DAQ device, and a personal computer (PC). A capacitance-type microforce sensor with a strain-inducing body (T130, Tech Alpha) was used with an amplifier (P210-N-130 Hz). The force range and the sensitivity were ±50 mN and about 10 *μ*N/mV, respectively. The output of the sensor was recorded via the DAQ device to the PC simultaneously with the signal for electrical stimulation. According to the sensor manufacturer, force and temporal resolutions are approximately 10 *μ*N (=1 mV) and 100 Hz, respectively. The noise level is less than 30 *μ*N (=3 mV), and hysteresis is negligible. The sensor was calibrated using gravity by hooking a bent thin metal wire of known weight on the sensor. During a measurement, a TEM was placed in the measurement chamber filled with Dulbecco's phosphate-buffered saline with calcium and magnesium (DPBS(+)), and the temperature of which was controlled by a custom-made temperature control unit using a Peltier element. The noise spectral density of the output was obtained by processing with Igor Pro (Ver. 7.0, WaveMetrics).

To avoid damage to the TEM, the TEM length should be kept as long as the initial state. Two assembly tools were made and used for the measurement. The first one was fabricated by a 3D printer (Aglista, Keyence), and it was able to hold the PTFE pillars by pinching two square holes with reverse action tweezers (Figure 2(b)). When the tweezers were open, the yellow and magenta part holes were overlapped perfectly and the PTFE pillars were released. When the tweezers were closed, the holes were partly overlapped and the PTFE pillars were locked into them. The reverse action tweezers which were closed in a free state were able to hold the TEM without any external force. Figure 2(c) shows the second tool which consisted of a PTFE sheet (2 mm thick) with two holes. These holes were tapered and PTFE pillars fit tightly in them so that the PTFE pillars were vertical and held the TEM stably.

The steps to position a TEM in the force measurement system are shown in Figure 2(d). First, a TEM was taken from the gel culture mold with the first assembly tool. Then, the TEM was transferred to the second assembly tool, and the tool was placed in the measurement chamber filled with DPBS(+). Finally, the TEM was removed from the assembly tool and placed on an attachment fabricated by the 3D printer. The TEM was fixed with M1.6 screws while observing it with two microscopes horizontally and vertically at the same time. Before the measurement, the length of the TEM was adjusted to the initial length (10 mm) using the manual stages.

Contractions of TEMs were evoked by field electrical stimulation. The signals generated from the function generator (FGX-2220, TEXIO) were amplified by the bipolar power supply. The contractile forces of twitch and tetanus were measured by applying mono polar pulses at 0.83 V/mm through the Pt electrodes. The electrical stimulation parameters for measurement were obtained from the literature [27]. The voltage was 24.9 V as the distance between the Pt electrodes was about 30 mm. For twitch contraction, a single monopolar pulse of 10 ms in width was applied. For tetanus contraction, monopolar pulses 10 ms in width at intervals of 10 ms were continuously applied for 2 s.

### 2.7. Histological Staining

After overnight fixation with 4% paraformaldehyde (163-20145, Wako Pure Chemical), TEMs were embedded in paraffin and sliced into a 4 *μ*m section. Then, the sections were stained with hematoxylin and eosin (H&E) and observed with a microscope (Ti-E, Nikon). The obtained images were combined by imaging software (NIS-elements, Nikon).

### 2.8. Statistical Analysis

Results were represented as mean values ± standard deviation. A comparison for all data was conducted with a Mann–Whitney *U* test, whereby *P* < 0.05 was considered as statistically significant.

## 3. Results

### 3.1. Microforce Measurement System for TEMs

**Figure 3:**
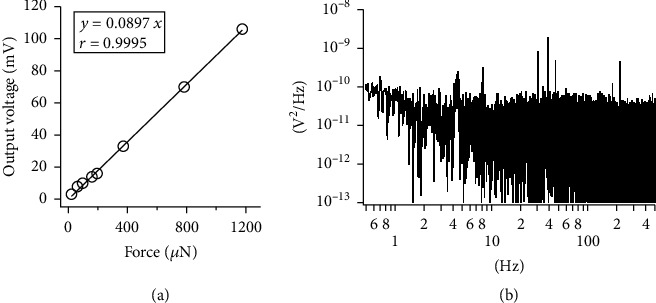
Evaluation of the force sensor. (a) Least squares fit of the calibration line. (b) Noise spectral density of the output.

We developed the microforce measurement system in order to measure the contractile force of TEMs under the isometric condition. The maximum contractile force of TEMs constructed by the present system could be assumed as 1 mN or more. The linearity of the system was examined in the range of about 1 mN.  Figure 3(a) shows a least-squares fit for the calibration line through the origin. The conversion factor was 11.15 *μ*N/mV and obtained as the reciprocal of the inclination. The output voltage when a TEM was attached to the force sensor fluctuated slightly due to electrical and mechanical noises. As shown in Figure 3(b), the noise spectral density analysis showed that the noise floor was around 10^−10^ V^2^/Hz. Several peaks over 10^−9^ V^2^/Hz were found around 4, 8, 30, 40, 50, and 200 Hz, which should be derived from the natural frequency of the mechanical system. These values indicated that the developed system was sensitive enough to measure the contractile force of TEMs.

### 3.2. Electrical Field Distribution in Gel Culture Chamber

**Figure 4:**
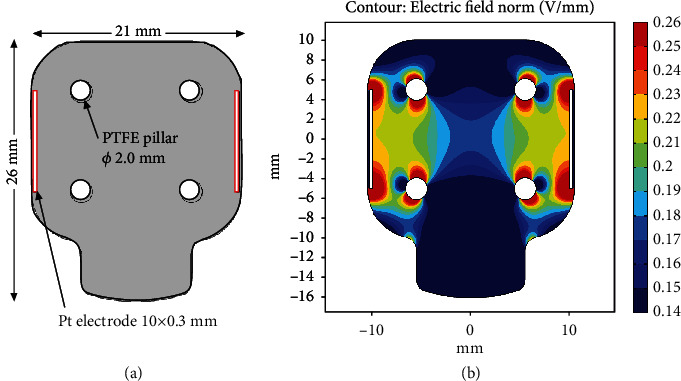
Simulation for electrical field distribution of the gel culture chamber. (a) Details of the 2D simulation model. The calculation was done only in the region in gray as the boundaries indicated in black were insulated. The electric potential on the surface of the Pt electrodes indicated in red was set at 4 V. (b) Contour plot of the electric field. The electric field around the area corresponding to the TEM was 2.0 V/mm or over.

It was necessary to confirm if the electrical field around the TEM was as strong as the simple calculation (applied voltage/distance between the electrodes) predicted since the shape of the culture chamber is complex compared to normal culture apparatuses and vessels. The electrical field distribution when 4 V was applied between the electrodes is shown in Figure 4. For most areas of the TEM, the electrical field ranged from 0.2 to 0.22 V/mm, which was consistent with 0.2 V/mm by a simple calculation (applied voltage divided by actual distance between the electrodes). Therefore, the electrical field applied to the TEM was considered to be the same as for the simple calculation. We noted that there were some high and low electrical field spots, over 0.25 V/mm and less than 0.15 V/mm, respectively, around the PTFE pillars due to PTFE being an insulator material. The center area where there was no TEM had a field of less than 0.18 V/mm and would not have any influence on TEM culturing.

### 3.3. Formation of Contractile TEMs without Electrical Stimulation

**Figure 5:**
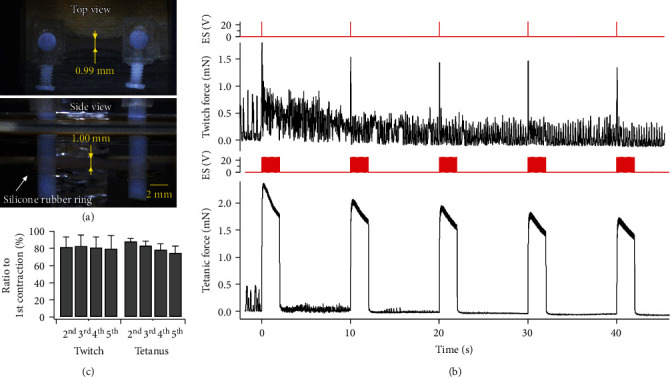
TEM cultured for 10 days without electrical stimulation. (a) Top and side views of the TEM are attached to the microforce measurement system. Silicone rubber rings were attached to the PTFE pillars so that the TEM would not be dropped. Due to cell-mediated gel compaction, the width and thickness of the TEM were reduced from 4 mm to 0.99 mm and 5 mm to 1.00 mm, respectively. (b) Representative twitch and tetanus contraction traces evoked by electrical pulses. (c) Declination of contractile force by repeated electrical stimulation. The force for each stimulation was shown as a ratio to the force for the first stimulation.

First, we confirmed that the system was able to produce TEMs without electrical stimulation for control. Although electrical stimulation was not used, the system cultured contractile TEMs. The TEMs were attached to the microforce measurement system using the assembly tools as shown in Figure 5(a). The TEM thickness could be compacted to less than 1/4 of the gel culture mold thickness. The average cross-section area of TEMs was calculated as 0.69 mm^2^ by assuming an ellipsoidal cross-sectional of TEM. Twitch and tetanic forces of contractions of TEMs under the stimulation conditions shown in Section 2.6 were measured for about 50 s (Figure 5(b)). The peak forces of twitch and tetanus (*n* = 5) had average values of 1.36 ± 0.21 mN and 1.93 ± 0.12 mN, respectively. The average specific forces for twitch and tetanus were calculated as 2.81 kPa and 3.66 kPa, respectively. The forces were comparable to TEM of C2C12 in some reports [37, 38].

As shown in Figure 5(b), the microforce sensor detected not only contractions responding to electrical stimulation but also spontaneous contractions of TEMs of about 0.5 mN. In particular, numerous peaks by the spontaneous contractions were found between twitch contractions. Intrinsically, skeletal muscles do not contract autonomously, while spontaneous and irregular weak contractions that occur without any stimulation are often found in well-differentiated myotubes [32, 34]. Spontaneous and repetitive firing in C2C12 myotubes has been reported to occur [39, 40], and it would be able to cause spontaneous contractions. The spontaneous contractions were at about 2 Hz before the first twitch. The frequency obviously increased after the first twitch contraction and decreased with increasing numbers of the twitch contractions evoked by electrical stimulation. On the other hand, the force of the spontaneous contractions decreased steadily. In addition, the spontaneous contractions drastically decreased after the first tetanus contraction. After the third tetanic contraction, hardly any spontaneous contractions were detected.

As shown in Figure 5(c), the peak contractile forces of the second and later contractions were smaller than that of the first one. The force of twitch contractions from the second stimulation was almost constant at around 80% of the first one. On the other hand, the forces in tetanic contractions gradually decreased from 85% to 75% of the first one as the stimulation was repeated. The results showed that repeated electrical stimulations lowered contractile forces of TEMs in spite of each one being a short-term stimulation.

**Figure 6:**
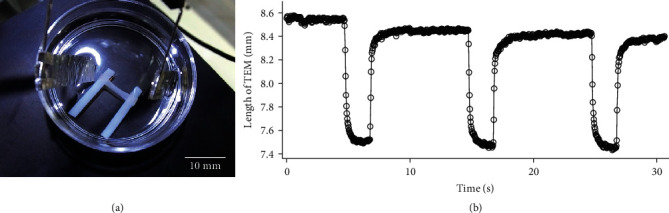
Image analysis of shrinkage of the TEM in a free state. (a) Application of continuous pulses to the TEM while still attached to PTFE pillars to evoke tetanic contraction. (b) Length trace of the TEM during electrical stimulation for tetanus.

The shrinking ability of the TEM was also confirmed by electrically stimulating the TEM in a free state for which the TEM length was not kept constant.  Figure 6(a) shows the experimental setup; the electrical pulses applied for the TEM were the same as for the force measurement of tetanic contractions. The TEM shrinking can be viewed in Movie S1.  Figure 6(b) shows the length obtained by image analysis using software (Dipp-Motion V, Ditect). The length was calibrated based on the length of the left pillar, which was 14.6 mm. The shrinkage distances of three contractions were nearly 1 mm, which was 10% of the initial length. It should be noted that the TEM was fixed at 10 mm during culturing and observations showed that shrinking began gradually as soon as the TEM was released, and the length was already less than 8.6 mm when the electrical stimulation started.

### 3.4. Effect of Electrical Stimulation on Contractile Force

**Figure 7:**
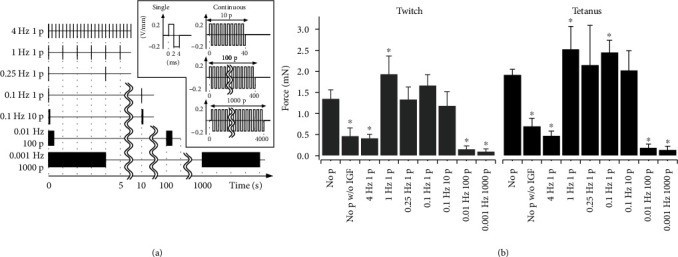
Contractile forces of TEMs cultured without and with electrical stimulation. (a) The patterns of the electrical pulses are applied to TEMs. The inset shows in detail single and continuous pulses. (b) Contractile forces of TEMs cultured under various conditions. For the case shown by the first bar from the left, the TEM was cultured without electrical stimulation but with the addition of IGF-1. For the case shown by the second bar, the TEM was cultured without electrical stimulation or addition of IGF-1. All other bars were for culturing cases with both electrical stimulation and IGF-1 addition. On the bottom axis, the frequency of the stimulation (Hz) is shown with “p,” which means the numbers of electrical pulses applied at one stimulus. The addition of IGF-1 increased the contractile force significantly, while the effects of electrical stimulation depended on the conditions. The ∗ indicates a statistically significant difference against “No p” in each group (*n* = 4 to 6).

To enhance the contractile force of TEMs, the effects of electrical stimulation on them were evaluated based on the contractile force. All the data for twitch and tetanus forces are summarized in Figure 7. Before the experiments with electrical stimulation, TEMs were produced without IGF-1 to evaluate the applicability of the culture system excluding the electrical stimulation function. IGF-1 is known to induce skeletal myotube hypertrophy [41], and it is often used to culture TEMs. Additionally, IGF-1 is known to increase the static tension and the contractile force of TEMs [24, 42]. As expected, the twitch and tetanic forces significantly decreased to 35% and 37% of the respective forces with IGF-1. The significant difference between TEMs with and without IGF-1 shows that the culture system including medium replacement function was able to consistently produce TEMs with high enough reproducibility to evaluate the effect of electrical stimulation.

Single electrical pulses were applied to TEMs at various frequencies. The electrical pulses 4 ms in width and 0.2 V/mm during culturing were chosen based on the previous study [27]. The detailed pattern of the single pulses is illustrated in Figure 7(a), and the contractile forces are shown by the 3rd to the 6th bars from the left in Figure 7(b). Both twitch and tetanic forces drastically decreased only at 4 Hz and significantly increased only at 1 Hz. The other pulses had no significant effect on the forces except for the tetanic force at 0.1 Hz. The results showed that suitable applied numbers of electrical pulses enhanced the contractile forces of TEMs while too many applications of the electrical pulses decreased the contractile force. The adverse effect on the TEMs should be due to electrochemical damage.

Next, electrical pulses were continuously applied to TEMs with the same total pulse numbers as for 1 Hz 1 p as follows: 10 continuous pulses every 10 s (0.1 Hz 10 p), 100 continuous pulses every 100 s (0.01 Hz 100 p), and 1000 continuous pulses every 1000 s (0.001 Hz 1000 p). As well as the single pulse pattern, these patterns are illustrated in Figure 7(a). These stimulations mimicked the stimulation to evoke the tetanus contractions. No significant increase was detected (7th to 9th bars from the left in Figure 7(b)), but continuous pulses > 100 drastically decreased the forces in spite of low frequency. As well as single electrical pulses at 4 Hz, continuous pulses also would cause electrochemical damage.

### 3.5. Morphological Comparison of TEMs

**Figure 8:**
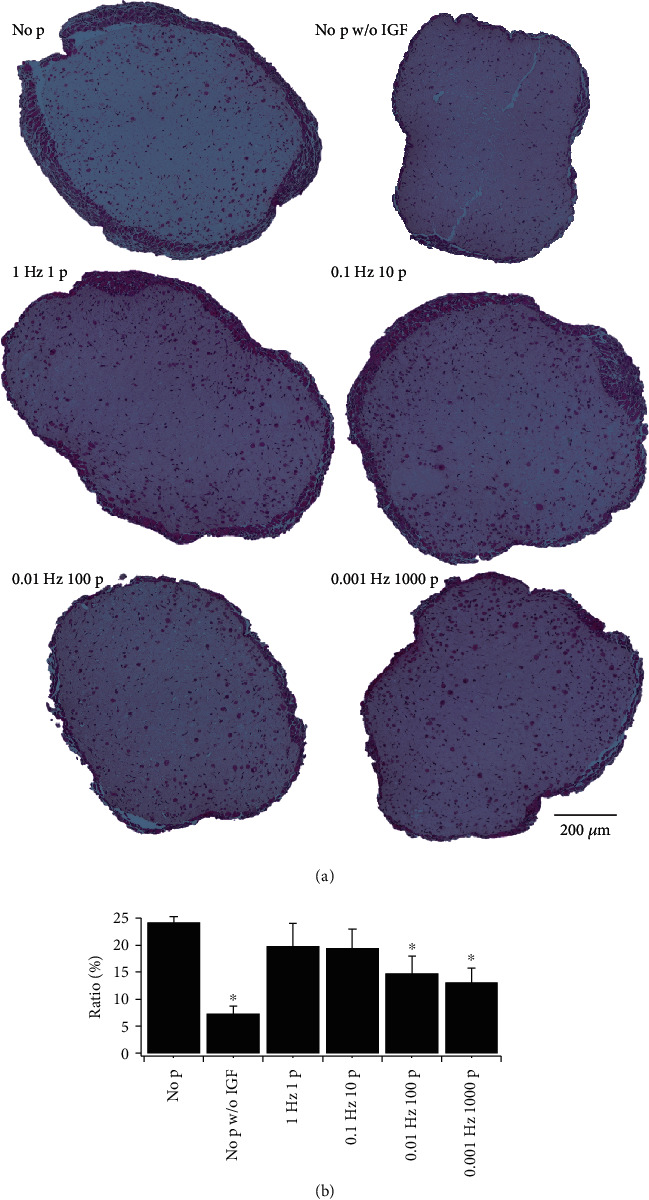
Comparison of cross-sections of TEMs. Culture conditions are labeled in the same manner as on the bottom axis of Figure 7. (a) Micrographs of HE-stained cross-sections. Deep purple dots show nuclei and areas in deep and light pink show cytoplasm and collagen gel, respectively. (b) Ratios of peripheral cell-rich regions to the whole. No electrical stimulation with IGF-1 had the highest ratio, almost 25%. The ∗ indicates a statistically significant difference against “No p” in each group (*n* = 4 to 6).

The effect of electrical stimulation on TEMs was also evaluated based on HE-stained cross-sections (Figure 8(a)). In all the cross-sections, muscle cells proliferated mainly in the peripheral regions, and fewer cells were present in the internal region. Clearly, TEMs of No p, 1 Hz 1 p, and 0.1 Hz 10 p had a thicker peripheral region than the others. The cell-rich regions within the whole TEM were manually recognized, and their ratios to the whole were calculated and plotted in Figure 8(b). No proportional relationship between the ratio of the peripheral region and the contractile force was detected, but the electrical stimulation seemed to decrease the ratio, namely, to inhibit cell proliferation. In spite of that, the TEM of 1 Hz 1 p demonstrated the strongest contractile forces, and its ratio was smaller than No p. Although Figure 7 showed that the appropriate electrical stimulation enhanced the contractile forces of TEMs, the result of Figure 8(b) suggests that electrical stimulation inhibits cell division.

## 4. Discussion

Low-labor production is one of the key technologies for practical use of biohybrid devices as well as traditional electric motors. We developed the daily maintenance-free culture system with the electrical stimulation and medium replacement functions, which could stably produce 12 contractile TEMs at the same time. To avoid ethical issues like using primary cells obtained by animal sacrifice, immortal myoblast cells were used. The system replaces GM or FM at the designated interval and also applies electrical pulses under the designated parameters. In particular, electrical stimulation can be applied to the TEMs in the same chamber where the cell-gel mixture is gelated.

Enhancing the contractile force of TEMs is also important for muscle-actuated devices. The TEMs produced without electrical stimulation generated tetanic contractions of almost 2.0 mN and were shortened by 1 mm. The most effective electrical stimulation (single pulses at 1 Hz) further increased the tetanic force over 2.5 mN. For instance, the actuator requirements for the reported TEM-actuated crawling robot [24] were estimated as contractile force of 1.5 mN and shortened distance of 0.15 mm, and the TEMs produced by our developed system are sufficiently able to satisfy those requirements. It should be noted that only tetanic contractions were focused on in the above discussion for the following reason. As tetanic contractions of skeletal muscles realize general motions in animals [43], tetanic contractions should be appropriate for utilization as an actuator.

The spontaneous contractions are not desirable for use as actuators since these contractions are not controllable. As shown in Figure 5(b), uncontrollable spontaneous contractions of TEMs were detected during the stimulation for twitch, but they were hardly ever detected for tetanus. Therefore, the spontaneous contraction should disappear with electrical stimulation to evoke tetanic contractions before using the TEMs. The spontaneous contractions of skeletal muscle cells and tissues cultured in vitro were reported by many groups [34, 44, 45], however, we could not find any study focused on spontaneous contractions. We think it likely that the continuous electrical pulses to evoke tetanic contractions caused electroporation of the cells slightly [45], which should temporarily lower their excitability.

In this study, the electrical stimulation was delivered to TEMs without a rest period, and single pulses 4 ms in width at 1 Hz were the most effective, which is basically consistent with previous studies [27, 45, 46]. In [27], the stimulation at 0.5 to 2 Hz increased the contractile force, and the stimulation at 1 Hz was most effective. In [46], more frequent stimulation even at 10 Hz did not cause damage to TEMs, however, the stimulation at 4 Hz in the present study seriously decreased the contractile forces. This should be because a rest period after the stimulation period was lacking (for example, 1 h stimulation, and 7 h rest periods); or the period for applying the stimulation (10 days in this study) was too long. Even a one-day stimulation period improved the contractile force of TEMs [45]. The continuous stimulation seemed to be harmful to TEMs in some cases, and the intermittent stimulation with a rest period would efficiently increase the contractile force of TEMs.

When the same total numbers of the pulses were applied as continuous pulses, the contractile forces were below those of the single pulses at 1 Hz. Even 10 continuous pulses at 0.1 Hz gave the same results as no electrical stimulation. On the contrary, continuous application of more than 100 pulses decreased the contractile forces below the case of no stimulation without IGF-1, which strongly suggests that the continuous pulses caused damage to TEMs. The cause of this damage should be direct electrical damage on the cell membrane like electroporation or damage caused by harmful byproducts of electrolysis like chlorine gas or hypochlorous acid. Further studies are necessary to clarify how continuous pulses have a bad influence on TEMs. The results on the contractile forces indicate that single pulses are appropriate at this time.

The HE-stained cross-sections revealed that the cells inside the TEMs did not grow under all the culture conditions that led to specific forces much lower than those of native tissues. This must be due to a lack of oxygen and nutrients supplies. Only Juhas and Bursac [47] achieved over 40 kPa of tetanic force by increasing the supplies using rocking culture, and this tetanic force was comparable with that of native neonatal muscles. Since TEMs are lacking for the circulation system inside like blood vessels and they are depending on supplies received only by diffusion, reduction of the size of the TEMs is the easiest way to distribute the cells densely and homogeneously. As well as rocking culture, medium agitation can deliver oxygen and nutrients to be consumed by TEMs [48]. Not only traditional techniques but also emerging one like microfluidics would be promising approaches to improve the forces of the TEMs. Taking into account the ratio of cell-rich regions as being around 20%, the contractile force will increase to around 10 mN if the myofibers are formed densely and homogeneously by resolving the issues of oxygen and nutrient supplies.

Other stimulations also might contribute to improved TEM production in the near future. It was reported that thermal stimulation at the stage of muscle bundle formation could increase contractile force at 39°C [38, 49]. On the other hand, mechanical stimulation has been used alone or with electrical stimulation to improve TEMs in studies that reported hypertrophy and increased protein and DNA contents and myotubes orientation in C2C12 muscle bundles [31, 50]. To the best of our knowledge, no study has been made to quantify the effect of mechanical stimulation on the contractile force of TEMs. As well as our developed daily maintenance-free culture system, the combination of static tension and electrical stimulation seems to work effectively to improve TEM production at the current stage.

This paper presented our daily maintenance-free culture system with electrical stimulation and medium replacement functions, by which we are working toward the long-term goal of practical use of muscle-actuated devices. This culture system could stably produce TEMs strong enough to be utilized for microrobots. The contractile forces of the TEMs were measured with our newly developed microforce measurement system. Even the TEMs cultured without electrical stimulation generated tetanic contractions of almost 2 mN and shortened lengths of 10%. Comparing the contractile forces, we saw that electrical stimulation by a single pulse at 1 Hz was most effective, and the contractile force in tetanus was over 2.5 mN. On the other hand, continuous pulses decreased the contractile forces. HE-stained cross-sections showed that oxygen and nutrients were not supplied to the interior of the TEMs sufficiently. By increasing the supplies, the TEMs would be able to generate a force up to around 10 mN. The daily maintenance-free culture system can surely contribute to the advancement of biohybrid devices by providing contractile TEMs at low-labor cost.

## Data Availability

The data used to support the findings of this study are available from the corresponding author upon request.

## Supplementary Materials

Supplementary Materials Movies S1: contracting TEM in a free state. Continuing monopolar pulses 10 ms in width at intervals of 10 ms for 2 s were applied to TEM through the Pt electrodes every 10 s.  Figure 6(b) was obtained by tracing the length of the TEM in this movie using image analysis.
